# Correction: Clathrin-independent pathways do not contribute significantly to endocytic flux

**DOI:** 10.7554/eLife.05256

**Published:** 2014-10-28

**Authors:** Vassilis Bitsikas, Ivan R Corrêa, Benjamin J Nichols

Bitsikas V, Corrêa IR Jnr, Nichols BJ. 2014. Clathrin-independent pathways do not contribute significantly to endocytic flux. *eLife* **3**:e03970. doi: 10.7554/eLife.03970

Published 17 September 2014

Four errors in the original figures were brought to our attention after publication. They arose inadvertently during assembly of the figures, which were prepared using Adobe Indesign. The boxes for display of multi-channel images were generated by duplication of a box already containing a linked image, and in some cases we failed to correctly update that linked image. We apologise for these errors.

1. In Figure 2A the image in the non-zoomed, greyscale panel of the streptavidin channel was duplicated in the transferrin channel. The zoomed images and the red/green overlay were correct.
2. In Figure 7G the image in the non-zoomed, greyscale panel of the streptavidin channel was duplicated from Figure 7E. The zoomed images and the red/green overlay were correct.
3. In Figure 2—figure supplement 3 the images in the streptavidin and transferrin channels were reversed.
4. In Figure 2—figure supplement 4 the image in the non-zoomed, greyscale panel of the transferrin channel was duplicated in the streptavidin channel. The zoomed images and the red/green overlay were correct.

Additionally, in the legend for Figure 3D, it was stated that ‘Bars are 10 µm’. This should have read ‘Bars are 20 µm’.

The article has been corrected accordingly.

**Figure 1.**
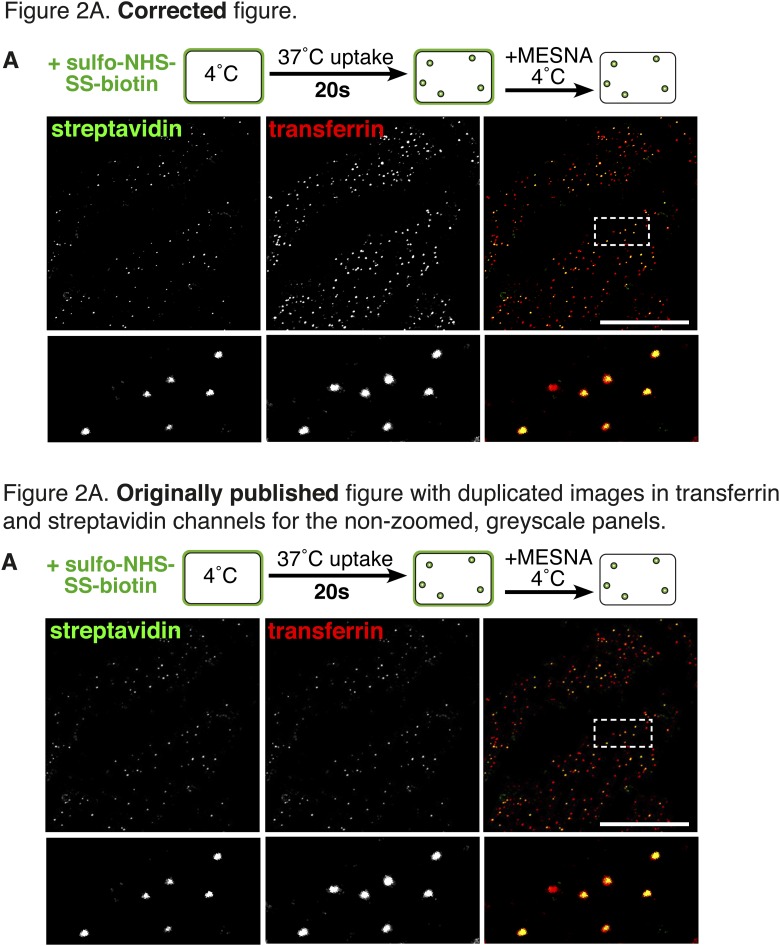
Corrections to Figure 2A.

**Figure 2.**
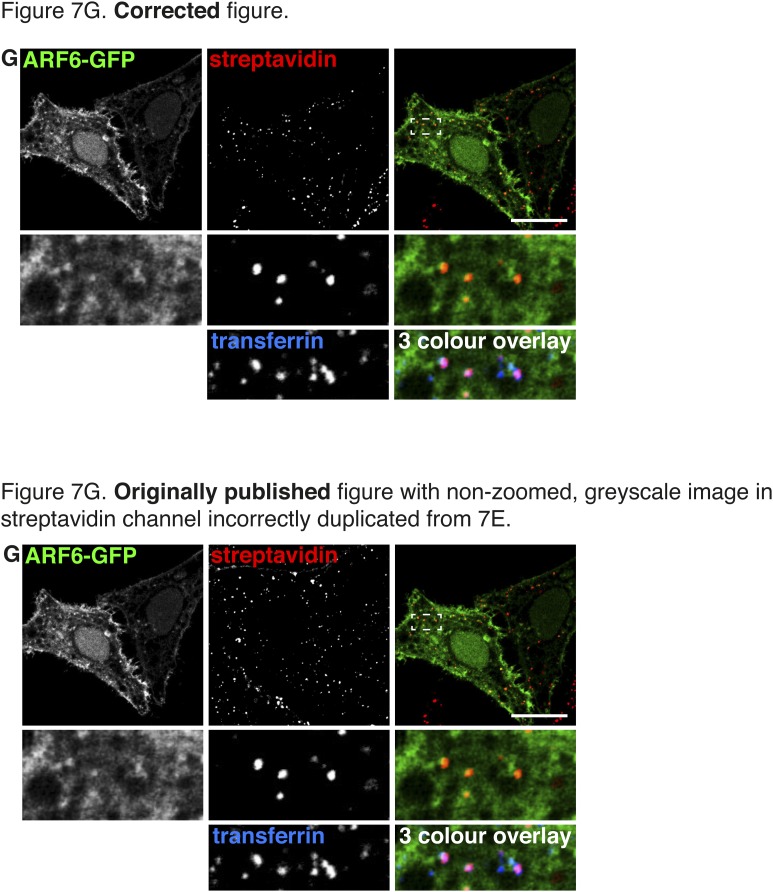
Corrections to Figure 7G.

**Figure 3.**
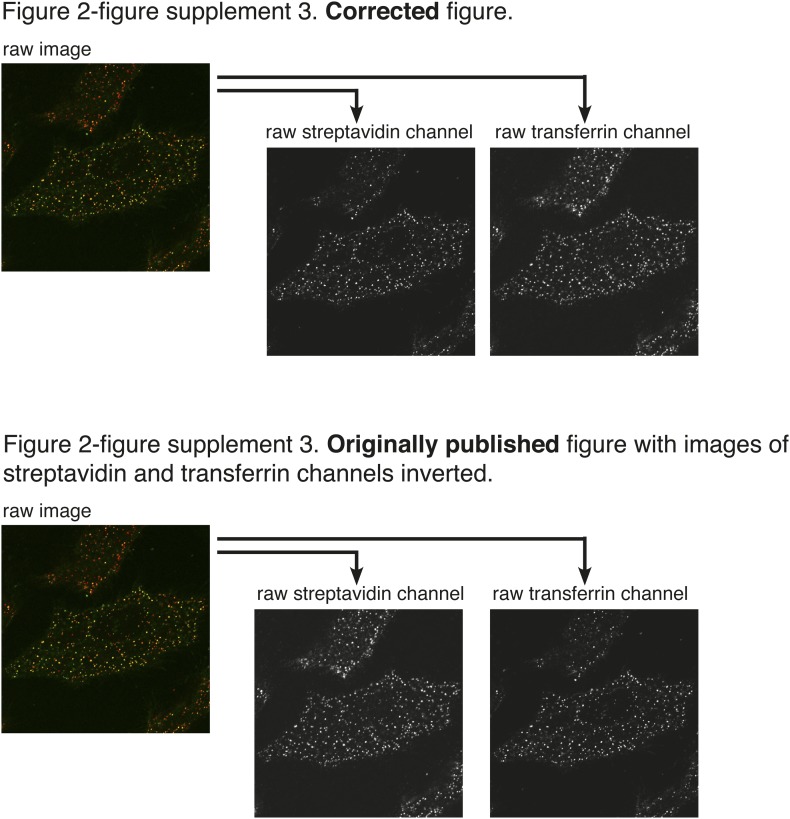
Corrections to Figure 2—figure supplement 3.

**Figure 4.**
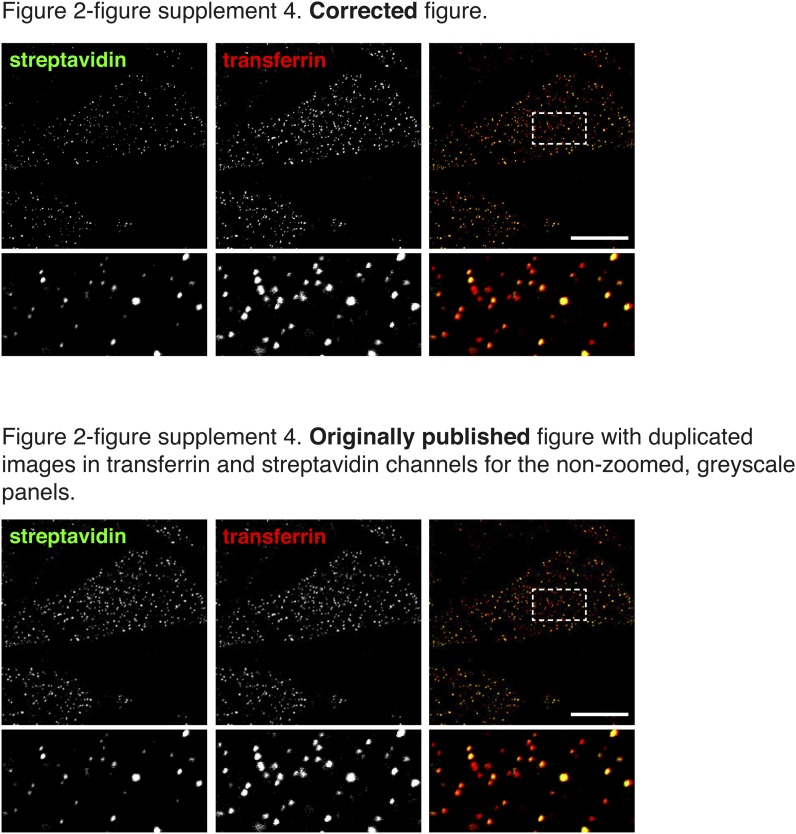
Corrections to Figure 2—figure supplement 4.

